# Responding to the Essential Sexual and Reproductive Health Needs for Women During the COVID-19 Pandemic: A Literature Review

**DOI:** 10.21315/mjms2021.28.6.2

**Published:** 2021-12-22

**Authors:** Mona LARKI, Farangis SHARIFI, Elham MANOUCHEHRI, Robab LATIFNEJAD ROUDSARI

**Affiliations:** 1 Student Research Committee, Mashhad University of Medical Sciences, Mashhad, Iran; 2 Nursing and Midwifery Care Research Center, Mashhad University of Medical Sciences, Mashhad, Iran; 3 Department of Midwifery, Faculty of Nursing and Midwifery, Mashad Medical Sciences, Islamic Azad University, Mashhad, Iran; 4 Department of Midwifery, School of Nursing and Midwifery, Mashhad University of Medical Sciences, Mashhad, Iran

**Keywords:** COVID-19, women’s rights, sexual health, reproductive health, reproductive right

## Abstract

The pandemic and its consequences have been shown to have a negative impact on the availability and accessibility of the basic services, including sexual and reproductive health (SRH) needs. The aim of this study was to investigate the responses to the essential SRH needs for women during the COVID-19 pandemic. This narrative review was conducted based on the Scale for Assessing Narrative Review Articles (SANRA), in order to present key activities for responding to the important SRH needs of women in the current COVID-19 pandemic. The literature search was performed through English databases of Cochrane Library, PubMed, Scopus and ScienceDirect, as well as Persian databases including Magiran and Scientific Information Database (SID). In addition, the World Health Organization (WHO), the Joint United Nations Programme on HIV and AIDS (UNAIDS) and the reports of Center for Disease Control and Prevention (CDC) were searched. The search was carried out from December 2019 up to 10 January 2021. Essential reproductive healthcare services for women during the COVID-19 pandemic were divided into six categories including access to contraception, safe abortion and post-abortion care, sexual health and sexually transmitted disease (STD) prevention, cervical cancer screening and prevention, maternity services and also addressing violence against women and girls. These essential needs should be considered as a priority by governments as well as public and private stakeholders. It seems that the implementation of the shifted strategies, active participation of public and private stakeholders, consideration of human rights and ethical issues, ensuring access to vulnerable populations, regular contact with individuals and the increased cooperation of individuals for responding to the SRH needs during the COVID-19 outbreak, are necessary.

## Introduction

The current COVID-19 pandemic is considered as the greatest health and socioeconomic crisis of our generation (1, 2). This pandemic and its consequences have negatively affected the availability and accessibility of the basic services, including sexual and reproductive health (SRH) needs (1, 3). However, the key sustainable development goals (3.7 and 5.6), in line with the criteria proposed by the Program of Action of the International Conference on Population and Development (ICPD) and the Beijing Platform for Action, have emphasised on ensuring all women’s equal access to SRH needs (4). In this situation, health systems around the world are threatened by the increasing demands for caring people with COVID-19, exacerbated by fear, stigma, misunderstanding and activity restrictions that subsequently disrupt the provision of healthcare services under all conditions. When healthcare services are overwhelmed and patients fail to receive the appropriate care, there is a dramatic rise in both direct mortalities resulted from an infection and indirect mortality resulted from preventable and treatable conditions (4).

Additionally, insufficient provision of essential services for SRH needs and, maternal and neonatal health during the COVID-19 pandemic would result in thousands of maternal and neonatal deaths, millions of extra unintended pregnancies, illegal abortions and complicated births (5, 6). It is noteworthy that, as estimated, a 6-month disruption of antiretroviral therapy (ART) in sub-Saharan Africa will result in over 500,000 adult deaths caused by HIV infection over a 4-year cycle and up to a twofold rise in mother-to-child transmission (MTCT) (4). Also, based on the degree to which health systems are affected and the extent of these delays, without applying any mitigation strategy, it is estimated that between 13 and 51 million women who may otherwise have used the current contraception, would be unable to do so in the future (7). Therefore, having access to comprehensive sexual and reproductive health and right (SRHR) needs, including clean and safe delivery, particularly for complicated pregnancies, treatment of sexually transmitted infections (STIs), safe abortion and performing post-abortion care, availability of contraception and provisions for clinical management of rape, is critical during pandemics (8, 9).

It should be noted that when access to healthcare services is expanded by some attempts to control outbreaks, care duties are also ‘downloaded’ to women and girls who are typically responsible for caring their family members as well as elderly people (8). Therefore, the risks of facing a pandemic are based on biological vulnerability, and social and economic effects of the infection. Moreover, it is expected that people’s perceptions will differ in terms of these determinants. As a result, the global and national strategic frameworks proposed for COVID-19 preparedness and response must be based on robust gender analysis, human rights-based approaches and ensuring the effective involvement of the affected groups, particularly women and girls, in both decision-making and implementation processes (10). Although the effects of the pandemic become worse, to control the transmission of the infection and restrict movements and shift resources to better respond to critical SRH needs, unprecedented steps are being taken by the governments and health system actors (1).

In this situation, the World Health Organization (WHO) suggested promoting virtual health services, self-care interventions, task sharing and community outreaches to assure people’s access to medications, diagnostic tests, as well as information and counselling services (4). In line with the WHO recommendation, health systems and police, judicial, and social care sectors in several countries have adapted their service delivery to the current situation by utilising the potential of internet and/or smartphone technologies, along with outreach groups to increase awareness and also to support survivors. In many settings, where United Nation Women (UNWomen) and the other international and national organisations have provided virtual services, some facilities such as phones, computers and the internet are not always available, particularly for lower-income or vulnerable community groups. Even when digital technologies are available, women rarely have access to these facilities or control over their use and may be carefully monitored by their partner or family members when doing so (11). Therefore, all adaptations should be made in line with some ethical values such as equality in resource sharing, access, self-determination, non-abandonment, and respecting dignity and human rights (12). The aim of this study was to investigate the responses of organisations and governments to the essential SRH needs for women during the COVID-19 pandemic.

## Methods

This narrative review was conducted based on the Scale for Assessing Narrative Review Articles (SANRA), in order to present key activities for responding to the important SRH needs of women in the current COVID-19 pandemic. Accordingly, SANRA is composed of the following six different items: i) justification of the article importance for the readership; ii) the aim of narrative review; iii) the detailed overview of the literature search; iv) referencing; v) incorporation of appropriate evidence and vi) appropriate presentation of data (13).

### Search Methods and Strategies for Identification of Studies

The databases searched for related literature included English databases of Cochrane Library, PubMed, Scopus and ScienceDirect, as well as Persian databases including Magiran and Scientific Information Database (SID). Additionally, relevant guidelines of WHO, the Joint United Nations Programme on HIV and AIDS (UNAIDS), the Center for Disease Control and Prevention (CDC) and the United Nations Population Fund (UNFPA) regarding SRH needs for women in the COVID-19 pandemic were also searched. Reference lists of the included studies were also reviewed to find additional references.

Keywords for the search included: ‘sexual health’, ‘reproductive health’, ‘sexual and reproductive health’, ‘sexual and reproductive rights’, ‘contraceptive methods’, ‘abortion’, ‘sexually transmitted disease’, ‘cervical cancer screening’, ‘maternity services’, ‘violence’ and their synonyms AND ‘COVID-19 pandemic’ or ‘coronavirus pandemic’. To increase precision in the search process, Boolean terms (AND/OR) were used to separate the keywords as well as medical subject headings (MeSH).

Type of study design was not restricted in order to obtain all the literature on SRH needs in COVID-19 pandemic. The search was performed from December 2019 up to 10 January 2021.

### Inclusion Criteria for Studies and Guidelines

- The studies provided evidence regarding SRH needs in the COVID-19 pandemic
- Guidelines related to the SRH needs in the COVID-19 pandemic

### Exclusion Criteria for Studies and Guidelines

- Having a language other than English or Persian
- Having no access to the full text of the article
- Published as a letter to editor or conference abstract

### Data Extraction and Management of Studies

At first, two authors (ML and FS) screened the titles and abstracts of all the retrieved documents (guidelines and studies), in order to check the inclusion criteria. They independently selected and then did the data extraction. In this regard, any inconsistency was discussed by a third author. There was no disagreement between the authors at this stage.

## Results

The essential reproductive health services were divided into the following six categories: access to contraception, safe abortion and post-abortion care, sexual health and sexually transmitted disease (STD) prevention, cervical cancer screening and prevention, maternity services, and also addressing violence against women and girls. Details of the potential impacts of COVID-19 on the essential SRH needs and key actions for responding to them are presented in Table 1.

### Access to the Contraception

It was estimated that about 47 million women are unable to access any modern contraception in 114 low- and middle-income countries if the typical lockdown or COVID-19-related interruption lasts for 6 months with some significant service disruptions (7). If the lockdown remains for every 3 months, up to 2 million additional women would be unable to use the current contraception, assuming the high rate of disruption in this regard (7). In these circumstances, the use of telephone and other digital technologies for screening, triage and referral for care; educating and advising where feasible; responding to concerns regarding the contraceptive method use, methods’ side effects management; continuing the use of the method to support clients; and providing information and access to short-acting methods such as condoms, pills and subcutaneous medroxy progesterone acetate, should be taken into account (14). In addition, pharmacies and drug stores should enabled to expand their various contraceptive products and to allow multi-month prescriptions and self-administration of injectable subcutaneous contraceptives, where accessible (4). Accordingly, developing tele-health systems for teenage individual counseling in terms of the principles of confidentiality and non-coercive decision-making, is recommended (14).

### Safe Abortion and Post-Abortion Care

It should be taken into account that 10% reduction in services could cause 15 million unintended pregnancies and 3.3 million unsafe abortions during the next 12 months, as estimated (6). Therefore, to prevent these cases, we should minimise physical visits to facilities and communications between clinicians and clients through the use of telemedicine and self-management techniques, where possible to ensure having access to a qualified physician if necessary (4). Telemedicine is a secure and private way used to have an early pregnancy abortion without the need for a doctor to be present. Correspondingly, it is appropriate for those who are self-isolating, women living in rural areas or whose commitments in childcare unable them to leave their home (15). To prioritise the use of medical abortion e.g. manual vacuum aspiration equipment to minimise clients’ waiting time in facilities (in favour of social distancing) as well as considering the possibility of abortion at home with misoprostol, if it is available and sufficient guidance (in order to harm reduction) is also accessible for clients (16).

### Sexual Health and STD Prevention

In sub-Saharan Africa, 25.7 million people infected with HIV and 16.4 million people taking ART are currently living at the higher risk of treatment interruptions due to the COVID-19 pandemic. Because HIV facilities are closed, supply chains are interrupted or staffs are overwhelmed (17). In order to achieve sexual health and STD prevention, the need for menstrual products should be firstly prioritised and then assured that they are included in the lists of the required priority health products, and the use of various devices to improve access to condoms and lubricants for healthy sexual activities (4). In times of isolation, it is vital to focus on proper messaging for having a safe and consensual intercourse (4). Additionally, the use of home-based self-testing for HIV and other STIs for the targeted population should be increased (4, 14).

### Cervical Cancer Screening and Prevention

During the current COVID-19 pandemic, essential initiatives including promoting self-sampling for HPV testing, facilitating specimen collection through pharmacies or drop-offs at health centres, and creating online counseling following a negative screening test, as well as appropriate care after a positive screening test should be provided (4).

### Maternity Services

It was reported that one in three women (33%) postpone or cancel their visit due to the pandemic. In addition, they may experience some problems with a healthcare provider for SRH needs and seeking birth control (18). Notably, access to women-centred, respectful professional services such as obstetric maternal screening tests, foetal medications, neonatal care, and mental wellbeing and psychosocial resources with readiness to care maternal and neonatal risks for pregnant women with suspected or confirmed COVID-19, are some of the general recommendations for maternity services (19). It is also required to give educational materials on basic hygiene practices related to COVID-19 to pregnant women and their families and to individualise the birth mode based on the women’s preferences and the obstetric indications (19). Triage and recommendations should also be provided on common discomforts, complaints or concerns and risks along with self-care advice (14). For home visits in childcare, it is recommended to make up tele-consultation for health promotion. Visiting sick children should also be considered as a priority with adequate IPC interventions (20). In addition, education is essential to minimise the anxiety of women regarding the effect of COVID-19 on pregnant women and newborns, and to promote the continued communication with healthcare services (20).

### Addressing Violence Against Women and Girls

Reports of the increased rate of domestic violence during the current COVID-19 epidemic have been published worldwide. This is because women and girls around the world are at risk for intimate partner violence, domestic violence, child abuse and other forms of gender-based violence when forced to stay at home for a long time (21). Two million female genital mutilation cases may occur during the next decade due to the pandemic-related disruptions in preventive efforts that would have been avoided on the other hand (7). Additionally, it may be due to an additional number of 13 million child marriages that may not otherwise have taken place between the years 2020 and 2030 (7). In this situation, it is important to identify and provide information on local resources for survivors (e.g. hotlines, shelters, rape support centres and counseling services), including their opening hours, contact information and whether services may be virtually delivered, as well as establishing referral linkages (22). Furthermore, caregivers and service providers should be aware of the risks and consequences of violating women (22).

## Discussion

Access to SRHR needs during the current COVID-19 outbreak is known as a major public health concern, which should be considered as a priority (23). The prioritising of the COVID-19 response by disrupting critical SRH need services is expected to increase the risk of both maternal and child death (24). In the present review, necessity of several important components in SRH needs and activities performed in several fields, including having access to contraception, safe abortion and post-abortion care, sexual health, STD prevention, cervical cancer screening and prevention, maternity services, and addressing violence against women and girls were highlighted. In addition, the need for these components has been verified in some previous studies conducted in this field. It is noteworthy that in relation to the previous humanitarian crises, Bloom-Feshbach et al. (25) in a research reported that the decreased access to family planning, abortion, prenatal care, HIV-related, gender-based abuse, and mental health services results in higher rates of both maternal and infant mortality. Moreover, Li et al. (26) in their study presented the preliminary evidence of interruptions in reproductive health services due to COVID-19 cases such as prenatal and postnatal assessment, delivery and abortion services, provision of contraceptives and management of STIs. Besides, even in a country with a sound drug supply system like China, contraceptives were out of stock or in a short supply in some regions during the current pandemic (26).

In the study by Lindberg et al. (18), the women mentioned that they had to postpone or cancel their SRH need treatment and raised doubts regarding their ability to afford or receive contraceptives. Even after removing all these stay-at-home restrictions, women might not pursue the requisite SRH need services because of having worries that they or a family member may be exposed to COVID-19 and providers may continue to provide reduced in-person treatment. Such interruptions in the necessary care might finally lead to poor results in SRH need treatment. However, according to the Eghtessadi et al. (27), the low level SRH need services results in COVID-19 call for civil society organisations, in order to enhance their demand for government accountability, which is compounded by pre-existing imbalances experienced by the marginalised groups. Although telemedicine may be regarded as a lifeline for resolving the acute crisis in SRH need services, which may be raised from this outbreak, there are still major obstacles regarding its optimal implementation, many of which will require policymakers and regulators to respond (28). A meta-analysis on the application and performance of telemedicine in Africa founded the use of telemedicine in South Africa and Ethiopia to some extent, but the restricted usage was observed in Nigeria and Burkina Faso due to insufficient political support (29). In line with other studies, it was shown that the expansion as well as the acceptance of innovative approaches to SRH need services such as telemedicine or the provision of contraceptives through email, may ultimately increase the access to support and also improve women’s SRH need status. Developing curricula for online sex education, their distribution and ensuring that both in-person and online instructions are required in response to school closures caused by pandemic, are necessary. Regardless of the form of healthcare delivery, the economic barriers generated by the loss of health insurance and declines in household income must be addressed to prevent aggravating existing inequities. Furthermore, we must ensure that all people have access to effective maternal, reproductive and sexual healthcare in a secure, free and dignified manner both now and also after the end of this pandemic. Additionally, the allocation of financial capacity and evidence-based interventions should be considered to deal with violence against women and girls during the COVID-19 pandemic. In addition, public health interventions to the pandemic must address the health needs of immigrants. It is notable that clinical services and facilities, monitoring and assessment research, advocacy and policy are all known as parts of the programmes in this context (2, 18, 21, 30, 31).

This study provided a broad overview of responding to the essential SRH needs for women during the COVID-19 pandemic. One limitation of our research was that quality assessment of the studies and guidelines was not performed. We reviewed studies published in English and Persian languages within the scope of this review and may therefore have excluded important data published in other languages.

## Conclusion

Although women should be delimited from attendance at health centres and exposure to healthcare providers according to the standards set out in the COVID-19 health protocols, they should not postpone their timely screening for diseases. Additionally, they should sustain continuing care for chronic conditions to prevent complications and exacerbations. In this regard, appropriate and practical, facility-based services should be provided online and primary care services that are routinely delivered over several visits, should also be combined if possible. Besides, clear information on when to seek care, how to receive services, payment methods and assurance about the safety of services should be provided as the part of the information plan for women. Lastly, it seems that the implementation of the shifted strategies, active participation of public and private stakeholders, consideration of human rights and ethical issues, ensuring access to vulnerable populations, regular contact with individuals and the increased cooperation among individuals for responding to SRH needs during the COVID-19 outbreak, are necessary, as well.

## Figures and Tables

**Table 1:** Potential impacts of COVID-19 on essential SRH needs and key actions for response

Essential SRH services	Potential impacts of COVID-19 on essential SRH services	Key actions for response to essential SRH needs
**Access to contraception**	An extra 7 million unwanted pregnancies are projected to occur if the lockdown lasts for 6 months and there are major service delays attributable to COVID-19 (7).	Recognise that the availability of contraceptives may decrease and therefore develop mixed contraceptive methods. Try to be able to change counselling programmes for family planning to redirect clients to alternative approaches (i.e. those that are different from the existing approach of a client). For example, clinicians may need to enhance counselling services in pregnancy awareness methods (such as the standard days method) and emergency contraception (16). Return to longer-term approaches (such as intrauterine devices [IUDs] and implants) and irreversible methods (tubal ligation and vasectomy) in the event of disruption of these programmes (4).
**Safe abortion and post-abortion care**	A 10% shift in abortions from safe to unsafe due to COVID-19 could result in 3,325,000 additional unsafe abortions and 1,000 additional maternal deaths (6).	Continue to provide facilities (medical and surgical) with quality abortion care (16, 32). In countries where legal, safe abortion facilities are constitutionally allowed, where non-urgent and elective services are suspended, safe abortion services must still be maintained (20). Adapt the forecast for products and services to accommodate the projected rise in medical requirements (4). In cases where women have access to reliable information and healthcare should they require or desire them at any stage of the process, women will securely self-manage surgical abortion (15).
**Sexual health and STD prevention**	A 6-month disruption of antiretroviral therapy due to COVID-19 in sub-Saharan Africa could result in over 500,000 additional deaths from AIDS-related diseases, including tuberculosis (17).	Ensure that clients under long-term treatment have sufficient access to essential commodities (e.g. HIV medications, menopause control or hormone therapy as part of gender-affirming care) (4). Prioritise, when practicable, screening of pregnant women for syphilis as part of routine antenatal care (ANC) (4). Modify the delivery of test results, care and preventive messages across digital platforms, including smart phones (4). Prioritise HIV tests for those at high risk, those with specified conditions (such as TB) and children with early childhood diagnosis (4).
**Sexual health and STD prevention**	A 6-month disruption of ART due to COVID-19 in sub-Saharan Africa could result in over 500,000 additional deaths from AIDS-related diseases, including tuberculosis (17).	Communicate on alternative, reusable products for menstrual hygiene. Involve community organisations, where available, to expand the distribution of menstrual products (3). Use mobile health approaches to deliver primary services and interact proactively with people with HIV where possible (14, 33).
**Cervical cancer screening and prevention**	Cervical cancer screenings decreased 94% between 20 January 2020 and 21 April 2020 (34).	Utilise a single-visit approach to screen for and treat precancerous lesions, if the capacity continues and facilities can be given safely (4). Prioritise access to screening for women living with HIV (4).
**Maternity services**	A modest 10% decrease in service coverage during pandemic could result in an additional 28,000 maternal deaths and 168,000 newborn deaths (4).	**ANC** Consider pragmatic reorientation of critical services, such as recognising ANC high-risk pregnancies and changing the timetable and making pre-appointments to health centres to minimise crowding and preserve physical distance (20). Replace ANC at health centres with home visits or tele-consultation to limit female exposure (20, 35). Prioritise ANC for high-risk pregnancy in health facilities and during the second half of pregnancy with appropriate infection prevention and control (IPC) interventions (20). Conduct clinical measurement (e.g. measuring blood pressure, fundal height, foetal heart rate, weight, as well as urinalysis) at any face-to-face contact (2, 36). **Postnatal care (PNC)** Consider rational improvements to services, such as prioritising first contact (within 24 h of delivery) and sufficient IPC guidelines (20). Distribution in accordance with national requirements of four PNC contacts (4). Replace additional communications with home visits, tele-consultation and counseling in no-risk situations (20). Consider providing long-acting reversible contraceptives in PNC (4). Make delivery of the recommended micronutrient supplements for 2–3 months (14).
**Maternity services**	A modest 10% decrease in service coverage during pandemic could result in an additional 28,000 maternal deaths and 168,000 newborn deaths (4).	**Home visits for childcare** Remind clients for social distance during the clinic session (e.g. standing two arms length away from each other) (20). Encourage breastfeeding mothers with COVID-19 symptoms and those with COVID-19 positive tests to adhere regularly to main protocols, including washing their hands and nipples before breastfeeding and using a face mask when feeding, in order to prevent transmitting the virus to their baby (16, 37). Encouragement for continued kangaroo mother care at home (14).
**Addressing violence against women and girls**	An extra 15 million additional cases of gender-based abuse are anticipated for every three months that the lockdown lasts (7). Thirty-one million extra events of gender-based abuse will be expected if the lockdown lasts for six months (7).	Be aware of the heightened risk of violence against women during this pandemic and of the need to keep in contact with and help women who are vulnerable to violence and to know where assistance is available to survivors (22). Ensure the post-rape care facilities are available, including emergency contraceptives, HIV prophylaxis and STIs care (4). Focus on improving survivor responses and provide help and support for their needs, including mental health and psychological support (4). Provide for women and families with economic and livelihood support (38). Enhance screening and programmes to detect abuse, gender-based violence (4). Locating safe houses, shelters or social service referrals for young people at risk of abuse in or near their homes (39).
